# Innovations in Pediatric Drug Formulations and Administration Technologies for Low Resource Settings

**DOI:** 10.3390/pharmaceutics11100518

**Published:** 2019-10-08

**Authors:** Stephen E. Gerrard, Jennifer Walsh, Niya Bowers, Smita Salunke, Susan Hershenson

**Affiliations:** 1 Global Health and Global Development Divisions, Bill and Melinda Gates Foundation, Seattle, WA 98109, USA; Niya.Bowers@gatesfoundation.org (N.B.); Susan.Hershenson@gatesfoundation.org (S.H.); 2 Jenny Walsh Consulting Ltd.; Nottingham NG1 1GF, UK; jenny@jennywalshconsulting.com; 3 School of Pharmacy, University College London, London WC1N 1AX, UK; s.salunke@ucl.ac.uk

**Keywords:** pediatric, drug development, formulation, global health, dispersible, suppository

## Abstract

Despite advances in regulations and initiatives to increase pediatric medicine development, there is still an unmet need for age-appropriate medicines for children. The availability of pediatric formulations is particularly lacking in resource poor areas, due to, for example, area-specific disease burden and financial constraints, as well as disconnected supply chains and fragmented healthcare systems. The paucity of authorized pediatric medicines often results in the manipulation and administration of products intended for adults, with an increased risk of mis-dosing and adverse reactions. This article provides an overview of the some of the key difficulties associated with the development of pediatric medicines in both high and low resource areas, and highlights shared and location specific challenges and opportunities. The utilization of dispersible oral dosage forms and suppositories for low and middle-income countries (LMICs) are described in addition to other platform technologies that may in the future offer opportunities for future pediatric medicine development for low resource settings.

## 1. Introduction

Pediatric patients are defined as patients aged from birth to less than 16 or 18 years, depending on region [1]. Due to development differences in physiology and cognitive and motor skills compared to adults, this population group often requires medicines that are tailored specifically for them. However, despite almost one third of the world’s population being less than 18 years old [2], there is a global lack of licensed medicines for pediatric use. This is due to factors such as the diversity in age and therapeutic needs of this patient population leading to greater challenges in conducting research into medicines for pediatrics compared to adult products. The availability of pediatric medicines is particularly lacking in resource poor areas because of a paucity of basic healthcare systems and fragmented supply chains, as well as mostly non-temperate and often harsh climatic conditions with a lack of refrigeration limiting formulation options, and area-specific disease burden creating a challenging market potential. This article provides an overview of the current difficulties and potential opportunities associated with the development of pediatric medicines for resource poor areas, with a focus on pharmaceutical development. Insights into the current situation regarding the formulation and administration of age-appropriate medicines for both high and low resource settings are provided to highlight shared and specific key challenges. The application of dispersible dosage forms, as advocated by the World Health Organization (WHO) is described [3], as well as suppositories and other platform technologies that may have the potential to provide opportunities for future development in low and middle-income countries (LMICs). (Defined as those with a gross national income per capita of less than $12,375 in 2018, in accordance with the World Bank [4]).

## 2. Background

Historically, there has been limited research and development into pediatric medicines, which has been in part due to a lack of market forces and economic benefits for the pharmaceutical industry compared to those for adult products [5]. This has been acknowledged and prompted global initiatives and legislative changes, with the aim of transforming this niche area into an integral part of the drug development process. The focus of these advances has been very much within the domain of high-income countries; regulations in the US and Europe [6,7,8] have made significant changes to the medicine legislature framework, and have led to an increase in the amount of information available on medicine administration to children, increased the number of authorized pediatric products and the number of new pediatric indications for already authorized products [5,9]. Progress is being made in other territories including China, where regulatory reforms have been designed to encourage the development of innovative drugs for pediatric patients in the future [10]. In addition, the Indian drug regulatory authority has acknowledged that pediatric regulation is undoubtedly needed [11,12], and the Indian Council of Medical Research have developed the National Ethical Guidelines for Biomedical Research Involving Children [13], to address ethical issues of conducting research in children. Almost every country in Africa has a national medicines regulatory authority; although they vary in terms of functionality and expertise [14]. Although specific pediatric regulations do not appear to have been adopted, The African Paediatric Fellowship Programme has been developed to provide relevant training for African child health professionals, by Africans, within Africa [15].

In 2007, the Member States of the World Health Organization (WHO) passed a resolution on “Better Medicines for Children” [16] and have since made progress on several fronts with the “Make medicines child size” initiative, including the development and publication of treatment guidelines, and information on the use of essential medicines such as the first Model List of Essential Medicines for Children and the WHO Model Formulary for Children [17]. Additionally, public–private partnerships, for example “Drugs for Neglected Diseases *initiative*” (DND*i*, https://www.dndi.org/), “Medicines for Malaria Venture” (MMV, https://www.mmv.org), and the “TB Alliance” (TBA, https://www.tballiance.org/) are addressing and promoting the development of pediatric formulations for global priority diseases.

Despite these developments, progress is slow and there is still a global scarcity of age-appropriate pediatric formulations, especially in LMICs, with disparities in life expectancy and burden of diseases [18,19]. There is an absence of specific pediatric development regulations in many resource poor countries [12,20] and financial constraints, workforce capacity and limited access to pediatric expertise have made it difficult for these countries to support new developments and maintain the high standards required.

A contributory factor in the lack of pediatric formulations worldwide and in LMICs in particular, may be due to the majority of therapeutic drugs (active pharmaceutical ingredients (APIs)) used to treat priority diseases being off-patent [21], since the investigation of off-patent pediatric medicines is voluntary, with potentially limited financial incentives to conduct this work [22]. Indeed, the paucity of authorized pediatric off-patent products has led to the prescribing of off-label/unlicensed medicines, whereby the medicine may be manipulated by healthcare professionals and caregivers to enable the intended dose to be administered to the patient [23,24,25,26,27,28,29,30]. The impact of such manipulations on the bioavailability and actual dose of API administered is often unknown, which can lead to an increased risk of medication errors and adverse reactions, as well as sub-optimal treatment [31,32,33,34,35].

## 3. Challenges for Progress and Innovations in Developing Pediatric Medicine Formulations

As stated above, pediatric patients have different requirements compared to adults regarding pharmacotherapy [36]. Key formulation parameters for the development of age-appropriate dosage forms regardless of the location of the proposed patient population include correct yet flexible dosing, acceptable excipients, easy product administration, and acceptable palatability. However, there are some differences in other factors between high and LMICs.

It is important that the optimal dose can be accurately administered to the patient, and hence the formulation may require some inherent flexibility to cover a range of doses. Although excipients are generally considered to be inert, new evidence suggests that there may be safety issues with some excipients when used in products for pediatric patients [36]. For example, immature metabolic systems in neonates and infants can lead to the accumulation and toxicity of propylene glycol [37] and benzoic acid/benzoates [38], and so these excipients should be used with caution in very young patients. Flavoring agents may be included to improve the taste of an oral product and facilitate patient adherence, but there are concerns regarding the potential risk of allergy and sensitization with these materials [39].

When formulating a pediatric medicine, it is also necessary to consider the capability of the patient to take the product and the product’s acceptability linked to achieving effective use, the latter being determined by the characteristics of both the product and the user (patient and caregiver) [36]. It is now increasingly acknowledged that the pharmaceutical development of a (pediatric) medicine involves more than its formulation aspects. For example, the recommended dosing frequency, type of packaging, type of medical device, or the comprehensibility of the user instructions may impact the medicine’s “intended use” in clinical and/or domiciliary practice [40]. In addition, the availability and ease of manufacture of the proposed dosage form should be borne in mind, since these will have an impact on cost and patient access. Indeed, patient acceptability, safety, and access must be balanced against each other, and a single “ideal” dosage form is unlikely to exist; in some situations, a compromise may need to be reached when selecting an age-appropriate formulation [41]. Additional challenges of developing pediatric medicines include the ethics and logistics of conducting trials in children. Challenges have been reported to arise from fears of harming children, political and economic influences, lack of resources, and a bureaucratic regulatory framework. In LMICs, there are also issues related to poverty, fear of participation, and mis-trust of sponsors [42].

Product attributes and supply logistics of pediatric medicines for resource-poor settings may differ compared to those of products designed for more economically developed countries, which can lead to additional formulation challenges, as summarized in Table 1. The climate in many LMICs requires products to be stable in high humidity and high temperature storage conditions (ICH Zones III and IV) [43], however, the provision of temperature-controlled storage and transportation in these areas is often limited and unlikely to be available in the near future. The use of solid dosage forms such as tablets is therefore preferred over liquids and semi-solids, since they are typically more stable [44]. Affordability is of key importance, since the majority of the population in developing countries may purchase their medicines through out-of-pocket payments [45]. Indeed, poor affordability can result in lack of availability of medicines in LMICs [46]. Although the cost of a product will depend on numerous factors, including the local pricing strategy applied and the utilization of inexpensive materials (including packaging), simple manufacturing and development processes will facilitate the implementation of low costs and greater patient access. Storage facilities and transportation systems in resource poor areas can be rudimentary and fragmented. Hence, any packaging should ideally be compact with a small footprint, light in weight, and sufficiently robust to withstand ground transportation in rural areas. For example, glass bottles are not a favored primary packaging option for global health products.

In addition to the above considerations, other aspects such as culture and health literacy need to be considered when defining the design criteria for the product. Medicine administration procedures and instructions should be simple to avoid misunderstanding. Caregivers may not understand information provided through healthcare workers, and use informal advice from immediate family members instead [47]. Non-adherence to medication is a global problem that is especially prevalent in LMICs, and factors reported to have a significant negative effect on adherence include poor socioeconomic status, poverty, low level of education, illiteracy, and long distance from treatment center [48]. Data from 2015 show that globally, 844 million people still lacked a basic drinking water service, with 159 million people still collecting drinking water directly from surface water sources, of which 58% lived in sub-Saharan Africa [21]. The potential lack of clean, filtered water can lead to difficulties in the constitution of powders and granules, and so, if this type of dosage form is developed, dispersal in other media such as breast milk may need to be considered.

## 4. Potential Dosage Form Opportunities for Low and Middle-Income Countries

In this age of advanced technologies, there has never been a greater opportunity to develop innovations for children in LMICs who remain vulnerable to the devastating effects of disease. Identifying technology platforms that would meet the needs of this pediatric population is a step towards addressing the lack of age-appropriate medicines. The dosage form examples highlighted below have been chosen to illustrate recognized technology platforms that have the potential to be applied to global health. It is acknowledged that the immunization of children against vaccine-preventable diseases can avert millions of deaths, as well as generate billions of dollars in economic benefits [49,50], and the same product development considerations highlighted above regarding acceptability, stability, cost, and supply should be considered. Specific vaccine technologies are also discussed in detail in various related recent reviews [51,52].

### 4.1. Oral Dispersible Dosage Forms

For oral products, the development of flexible solid dosage forms, for example tablets that can be dispersed or dissolved in a beverage prior to administration, have been recommended by WHO [44]. They are considered to be suitable for LMICs because they have some of the advantages of solid oral dosage forms, such as superior stability and less bulk compared to liquids, and once dispersed are acceptable for patients of all ages, including those who have difficulty in swallowing tablets. A summary of the potential advantages and disadvantages of dispersible dosage forms is provided in Table 2.

**Figure 1:**
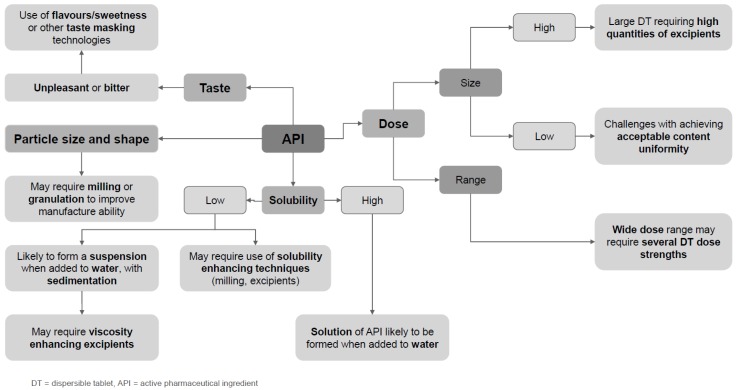
Map of factors of active pharmaceutical ingredients (API) affecting dispersible tablet formulation approaches.

Various API properties need to be considered when developing dispersible dosage forms (see Figure 1).

For example, the dose of API will affect the unit dose quantity of granules or overall size and dimensions of the dispersible tablet, and hence the volume of liquid required for complete dispersion. API physicochemical properties such as particle size and morphology will impact the manufacturing process and choice of excipients. The aqueous solubility of the API will affect how the granules or tablet disperses in liquid, and if a solution or a suspension of the API is formed; if the API fully dissolves, the need to rinse the vessel or add thickeners to the formulation to reduce sedimentation may be avoided. Solubility can affect the bioavailability of an API, and so also has an impact on required dose. APIs with an unpleasant taste are likely to require a sweetener and/or flavor to improve palatability and ensure patient compliance, whilst additional taste-masking techniques may be needed for very aversive APIs [41].

Despite some of the challenges highlighted above, several dispersible tablet formulations have been successfully developed for pediatric patients in LMICs. Examples are provided in Table 3.

### 4.2. Rectal Forms

Administration of drugs via the rectum offers an alternative to the oral route and is suitable for both local (for example, constipation, hemorrhoids, analgesia) and systemic delivery (for example analgesia, infections, and management of central nervous system disorders), and minimizes hepatic first pass metabolism, since the API is absorbed directly into the systemic circulation, thereby avoiding the liver. Indeed, the extent of rectal absorption has been reported to exceed oral values for some APIs [53]. Dosage forms for the rectal route such as suppositories are suitable for pediatric patients who may have difficulty swallowing tablets and capsules. Furthermore, they do not require taste-masking and can be administered to unconscious or vomiting patients [54]. Suppositories (solid dosage forms that dissolve or melt when inserted into the rectum) constitute most current products administered rectally in clinical practice. They offer several advantages compared to other dosage forms, especially in LMICs. For example, they might be used for the treatment of children in rural areas where parenteral administration is not possible. A summary of the potential advantages and disadvantages of suppositories is provided in Table 4.

There are two main types of suppository bases; fatty bases that melt at the temperature of the rectum, and water-soluble bases that dissolve in the rectum [55]. The selection of base depends on the properties of the API, for example, water-soluble APIs tend to be formulated in fatty bases and lipophilic APIs in water-soluble bases. As with dispersible dosage forms, the required dose of API will impact the size of the suppository. However, age-related changes in the dimensions of the rectum need to be considered to ensure acceptable administration. Although suppositories are considered to be appropriate for children [56], a key constraint in their use appears to be the varying levels of their acceptability around the world and according to patient age. This is an area in which patients and caregivers require further education.

Several new suppository developments are on-going to mitigate some of the disadvantages highlighted above, for example, the use of muco-adhesives and high melting point excipients to improve bioavailability and heat stability, respectively [54]. Despite the above challenges, and potential for regionally specific acceptability, the development of suppositories for LMICs is an area of growing interest [57]. For example, suppositories containing 100 mg of artesunate have been developed and prequalified by WHO for pre-referral treatment for patients aged between six months and six years, with suspected moderate or severe malaria who are unable to take oral medication or obtain injectable antimalarial treatment [58].

## 5. Other Potential Formulation Technologies for Low and Middle-Income Countries

There are other formulation technologies in addition to dispersible dosage forms and suppositories that may also be in the future applicable to pediatric patients in LMICs. For example, mini tablets (1–3 mm in diameter), like other solid oral dosage forms, have the advantages of better stability and less bulk compared to liquids, but unlike standard tablets, have been reported to be acceptable from birth if uncoated and from six months if coated [59,60]. Mini tablets are a relatively recent innovation, and hence few products appear to have been commercialized, and none specifically for LMICs. Examples of marketed products include Orfiril^®^ long (sodium valproate sustained release), LAMISIL^®^ oral granules (terbinafine hydrochloride), KALYDECO^®^ oral granules (ivacaftor) and Desitin^®^ minitablets (levetiracetam). Oro-dispersible tablets (ODTs) are designed to disperse in the oral cavity without the need for a beverage and are also considered be acceptable for young patients [61], although may require moisture protective packaging. Several ODT products are commercially available, although the majority are only licensed for adult use. Examples of pediatric ODT products include Loratidine ODTs, Calpol^®^ Six Plus Fast Melts (paracetamol), and Nurofen^®^ for Children Meltlets (ibuprofen).

Transdermal delivery, where APIs are delivered through the skin, has several advantages including the avoidance of first pass metabolism, controlled release, and potentially improved patient compliance. However, due to the barrier properties of the skin, only a few APIs can be delivered via this route at therapeutic levels. Microneedles are being developed to address this issue whereby micron sized pores are created through which APIs and vaccines may be administered. The microneedles are long enough to penetrate the dermis but avoid stimulation of dermal nerves and do not puncture the dermal blood vessels [62,63]. This is an emerging technology for which no drug delivery products appear to have yet been marketed, although numerous clinical studies have been conducted or are on-going [64]. Microneedles may offer advantages for LMICs, since it is an easy to use technology which is less invasive and more affordable compared to parenteral delivery [65].

## 6. Conclusions

The development of pediatric medicines has greater challenges compared to the development of medicines intended for adult patients, which has led to a significant unmet medical need. Additional considerations need to be applied to the development of pediatric medications for LMICs, including those linked to cost, acceptability, usability, heat stability, healthcare provider training, health policy, and local regulatory requirements. Although progress is being made, there is still a paucity of available age-appropriate pediatric medicines in LMICs. A number of technology platforms including emerging technologies which may offer potential additional solutions to mitigate some of the challenges associated with the development and supply of pediatric products for LMICs. The global health community should maximize opportunities to address the disparity in access to medicines that meet the needs of neonates, infants and children, where there is a great opportunity on long term health impact.

## Figures and Tables

**Table 1:** Exemplative comparison of potential considerations for pediatric medicine, pharmaceutical development, and supply requirements for high income economies compared to low- and middle-income economies.

Property	Traditional Pharma Drug Development	Global Health Drug Development	Impact on Development Opportunities for Low Resource Settings
Target population	0–18 years	0–18 years	None
Excipients (safety)	Acceptable for proposed patients	Acceptable for proposed patients	None
Acceptability	Palatable, non-irritant	Palatable, non-irritant	Minimal. Potential cultural differences (e.g., flavors) may need to be considered
Dose preparation	Minimal manipulation/preparation, oral products may be mixed with water/food/beverage	Minimal manipulation, preferably no preparation	Avoid requirement for mixing if possible. If required, consider readily available vehicles, e.g., breast milk
Administration	Easy to administer, use of administration device if necessary	Easy to administer, preferably no administration device needed	Consider alternative options for dosing (no device)
Storage conditions	25 °C–30 °C/60–65% RH, refrigerated accepted (2–8 °C)	30 °C/75% RH	Ready to use liquids and semi-solids less favorable
Packaging	Various, no restrictions	Compact, light in weight, robust	Select light/compact container closure (preferably not glass)
Cost	Low overall cost ideal but not necessary	Low – standard non-complex manufacturing with low cost raw materials	Select only routine processes and commonly available, non-specialist, low cost excipients
Supply chain	Generally well-developed and efficient	Poorly developed and fragmented	Longer shelf life may be required, robust packaging
Regulatory	Mature and well-recognized regulatory requirements	Disparate regulatory requirements	Tailor regulatory strategy to each country/market

**Table 2:** Advantages and disadvantages of oral dispersible dosage forms.

Advantages	Disadvantages
**Dosing**	
Once dispersed in liquid is easy to swallow; suitable for the whole pediatric population from birth upwards	Limited dose flexibility, although a break line may be introduced to sub-divide tablets; granules generally provided in unit-dose packs (e.g., sachet); more than one dose strength may be needed to cover the required dose range
**Administration**	
Generally non-complex and simple method of administration, with no need for measuring device (e.g., dosing cup, spoon, or oral syringe)	Requires dispersion in water or other beverage prior to administration; whole volume of liquid dispersion must be taken; rinsing of vessel may be required to ensure all residue (if any) is taken
**Excipient Safety**	
Do not require the inclusion of preservatives; many excipients commonly used in dispersible dosage forms have an acceptable safety profile in pediatric patients	May require sweetener and/or flavor to ensure acceptable palatability
**Stability**	
Better stability than liquids or semi-solids	May need moisture protective packaging; in-use stability once dispersed likely to be limited; compatibility with dispersing vehicle should be confirmed
**Manufacture and Supply Chain**	
Non-complex development process; standard manufacturing and packaging equipment may be used; low bulk/footprint; easy to store and transport	Humidity control may be required during manufacture

**Table 3:** Examples of Dispersible Products for Low to Middle-Income Countries.

Product Name	API and Strength	Indication
Coartem^®^ Dispersible	Artemether 20 mg/Lumefantrine 120 mg	Uncomplicated malaria due to *Plasmodium falciparum.*
SPAQ-CO	Amodiaquine 150 mg Sulfadoxine-Pyrimethamine 500 mg/25 mg	Seasonal malaria chemoprevention
Paracetamol Dispersible tablets	Paracetamol 100 mg and 250 mg	Pain
Zinc Dispersible tablets	Zinc 20 mg	Diarrhea
Amoxicillin Dispersible tablets	Amoxicillin 125 mg and 250 mg	Pneumonia
Sulfamethoxazole/Trimethoprim Dispersible tablets (cotrimoxazole)	Sulfamethoxazole 100 mg/Trimethoprim 20 mg	Pneumocystis pneumonia, prophylaxis against infections in HIV patients
Lamivudine/Nevirapine/Zidovudine 30/50/60 mg dispersible tablets	Lamivudine 30 mg/Nevirapine 50 mg/Zidovudine 60 mg	Treatment of HIV-1
Lamivudine/Stavudine/Nevirapine	Lamivudine 30 mg/Stavudine 6 mg/Nevirapine 50 mg and Lamivudine 60 mg/Stavudine 12 mg/Nevirapine 100 mg	Treatment of HIV

API: Active Pharmaceutical Ingredient; HIV: Human Immunodeficiency Virus.

**Table 4:** Advantages and disadvantages of suppositories.

Advantages	Disadvantages
**Dosing**	
Suitable for pediatric patients from one month and unconscious or vomiting patients; able to deliver high doses of API; generally avoids first pass metabolism; can be used for local or systemic delivery; suitable for APIs that are gastro-irritant or prone to degradation in the stomach	May be associated with variable API absorption; potentially reduced API absorption if rectum is not empty; not recommended in preterm neonates due to risk of trauma and resulting infection; limited dose flexibility and more than one dose strength/size may be needed to cover the required dose range
**Administration**	
Generally non-complex method of administration, although training may be required to ensure correct insertion; no need for administration device although some devices are available (e.g., suppository inserter)	Route of administration may not be acceptable to some patients/caregivers (social/cultural reasons), leading to non- compliance; suppository may be expelled (involuntarily or via defecation)
**Excipient Safety**	
Do not require the inclusion of sweeteners or flavor; many excipients commonly used in suppositories are well tolerated by the rectal mucosa and have an acceptable safety profile in pediatrics	Some excipients may cause mucosal irritation
**Stability**	
	Can melt at temperatures above 30 °C
**Manufacture and Supply Chain**	
Relatively low-cost excipients; lower bulk/footprint compared to liquids; easier to transport than liquids	Manufacture more difficult than other common dosage forms (tablets, liquids); may need temperature-controlled storage (depending on melting point); humidity control may be required during manufacture
